# Life and Death of mRNA Molecules in *Entamoeba histolytica*

**DOI:** 10.3389/fcimb.2018.00199

**Published:** 2018-06-19

**Authors:** Jesús Valdés-Flores, Itzel López-Rosas, César López-Camarillo, Esther Ramírez-Moreno, Juan D. Ospina-Villa, Laurence A. Marchat

**Affiliations:** ^1^Departamento de Bioquímica, CINVESTAV, Ciudad de Mexico, Mexico City, Mexico; ^2^CONACyT Research Fellow – Colegio de Postgraduados Campus Campeche, Campeche, Mexico; ^3^Posgrado en Ciencias Genómicas, Universidad Autónoma de la Ciudad de México Ciudad de Mexico, Mexico City, Mexico; ^4^Escuela Nacional de Medicina y Homeopatía, Instituto Politécnico Nacional Ciudad de Mexico, Mexico City, Mexico

**Keywords:** *Entamoeba*, mRNA decay, mRNA processing, P-bodies, polyadenylation, protozoan parasite, splicing

## Abstract

In eukaryotic cells, the life cycle of mRNA molecules is modulated in response to environmental signals and cell-cell communication in order to support cellular homeostasis. Capping, splicing and polyadenylation in the nucleus lead to the formation of transcripts that are suitable for translation in cytoplasm, until mRNA decay occurs in P-bodies. Although pre-mRNA processing and degradation mechanisms have usually been studied separately, they occur simultaneously and in a coordinated manner through protein-protein interactions, maintaining the integrity of gene expression. In the past few years, the availability of the genome sequence of *Entamoeba histolytica*, the protozoan parasite responsible for human amoebiasis, coupled to the development of the so-called “omics” technologies provided new opportunities for the study of mRNA processing and turnover in this pathogen. Here, we review the current knowledge about the molecular basis for splicing, 3′ end formation and mRNA degradation in amoeba, which suggest the conservation of events related to mRNA life throughout evolution. We also present the functional characterization of some key proteins and describe some interactions that indicate the relevance of cooperative regulatory events for gene expression in this human parasite.

## Introduction

The metabolism of messenger RNA (mRNA) is a complex process that is essential for gene expression regulation and mRNA turnover in response to environmental signals and cell-cell communication in eukaryotic cells. During pre-mRNA synthesis by RNA polymerase II (RNA Pol II) in the nucleus, they are modified to generate mature transcripts that can be exported to the cytoplasm and translated to proteins. First, the 5′ end of nascent mRNA is capped by a 7-methyl guanosine linked by a 5′-5′ triphosphate bridge to the first nucleoside of the transcript (capping). These reactions are catalyzed by three enzymes: RNA triphosphatase, guanylyltransferase, and RNA (guanine-7-)-methyltransferase (RNMT) (Cowling, 2010). Then, introns are removed and exons are ligated by the catalytic activity of the spliceosome components that include five small nuclear RNA (snRNAs), namely U1, U2, U4, U5, and U6, and small nuclear ribonucleic proteins (snRNPs) (splicing) (Shi, 2017). Finally, a phosphodiester bond is hydrolyzed at the 3′ end of mRNA and a poly(A) tail is added by the coordinated activity of a large set of polyadenylation factors that recognize specific motifs in RNA 3′ untranslated region (3′UTR) (cleavage/polyadenylation) (Xiang et al., 2014). After translation, the elimination of mRNA molecules is necessary to ensure proper course of gene expression and prevent the accumulation of transcripts (Christie et al., 2011). Pathways of mRNA decay depend on the formation of RNA-protein complexes in microscopically detectable cytoplasmic structures, called processing bodies (P-bodies) (Sheth and Parker, 2003), in which mRNAs are translationally repressed (silenced) or degraded; their re-incorporation into ribosomes is also possible (Eulalio et al., 2007). Transcript decay involves 3′ end deadenylation by CAF1 and CCR4/NOT1–5 complex (or by PARN, PAN2 and PAN3 deadenylases) followed by 5′ end decapping by DCP1–DCP2 complex and Lsm1–7 proteins, and 5′-3′ digestion by exonuclease XRN1; alternatively, deadenylated transcripts can be degraded from the 5′ end by the exosome complex, while the scavenger-decapping DCP enzyme hydrolyzes the remaining cap structure (Łabno et al., 2016). During translation, aberrant mRNAs with premature termination codons can be detected and eliminated through the nonsense-mediated decay (NMD) pathway (Rebbapragada and Lykke-Andersen, 2009).

Although pre-mRNA processing reactions have usually been studied separately, they occur co-transcriptionally, simultaneously and in a coordinated manner. Moreover, a large set of data has shown that they are interconnected with transcription, translation, and mRNA degradation; protein-protein interactions establish a functional link between the different molecular machineries and promote reciprocal regulation events to maintain the integrity of gene expression. Consequently, each of these processes plays a major role throughout the life cycle of mRNA. Thus, in addition to protect mRNA from 5′ to 3′ exonuclease cleavage, the m7G cap interacts with the cap-binding complex (CBC), which regulates spliceosome assembly, transcription termination, 3′ end processing, RNA export, and NMD in the nucleus. In the cytoplasm, CBC recruits eIF4G, RNA helicase eIF4A, and other proteins to promote translation initiation. Moreover, eIF4G interacts with poly(A) binding protein PABP1 bound to the poly(A) tail to create a mRNA pseudo-circularization and enhance the processivity of the ribosome. Furthermore, it has been recently demonstrated that 2′O methylated cap (cap 1) acts as a signature of self RNA molecules (Ramanathan et al., 2016). Several data indicate that U1 snRNP, the more abundant splicing factor, inhibits 3′ end processing. Notably, its interaction with PAP inhibits poly(A) tail synthesis and promotes degradation of U1A pre-mRNA (Gunderson et al., 1994, 1997). Moreover, its binding to the 5′ splice site (5′ss) of the terminal intron, avoids the use of premature cleavage and polyadenylation to protect the integrity of the transcriptome (Furth et al., 1994). Other data indicate that splicing and 3′ end processing factors may recruit each other and form a stabilized complex on the target pre-mRNA, resulting in reciprocal stimulation of efficiency. Thus, interactions between U2AF65 and CFIm59 (Millevoi et al., 2006), or U1A and CPSF160 (Lutz et al., 1996), enhance the polyadenylation reaction, while CPSF (Kyburz et al., 2006) and PAP (Vagner et al., 2000) stabilize U2AF65 to the terminal intron to stimulate splicing. On the other hand, exon–exon junction complexes (EJC) participate in mRNA degradation, as part of the CBC whose CBP80 component interacts directly with the NMD factor, up-frameshift 1 (UPF1), enhancing the efficiency of this process (Isken and Maquat, 2008). CFIm may bridge 3′ processing with capping through the binding of CFIm25 with CBP20 (Yang et al., 2011).

Until recently, little was known about mRNA metabolism in *Entamoeba histolytica*, the protozoan responsible for human amoebiasis. The availability of the *E. histolytica* genome sequence and the development of the so-called “omics” technologies have provided new opportunities for the study of mRNA processing and turnover in this parasite. To our knowledge, capping has not been described in *E. histolytica*, although preliminary searches in parasite genome database suggest the presence of genes that encode proteins with similarities to human capping enzymes. In this review, we focus on the current knowledge about the molecular basis for splicing, 3' end formation and mRNA degradation, and describe some interactions between these events.

## What is known about splicing in *E. histolytica*

### Splicing factors

There are nearly four thousand introns in the 8333 annotated genes of *E. histolytica* (Weedall and Hall, 2011), most of them flanked by highly conserved 5′ and 3′ splice sites (ss), GUUUGU and UAG, respectively, but their branch point sequences (BS) lack such degree of conservation (Wilihoeft et al., 2001; Hon et al., 2013). Whereas, no minor U12 introns have been identified in amoeba and most likely neither in the eukaryotic ancestor (Collins and Penny, 2005; Bartschat and Samuelsson, 2010), the majority of the main spliceosome components have been predicted and identified.

**Figure 1:**
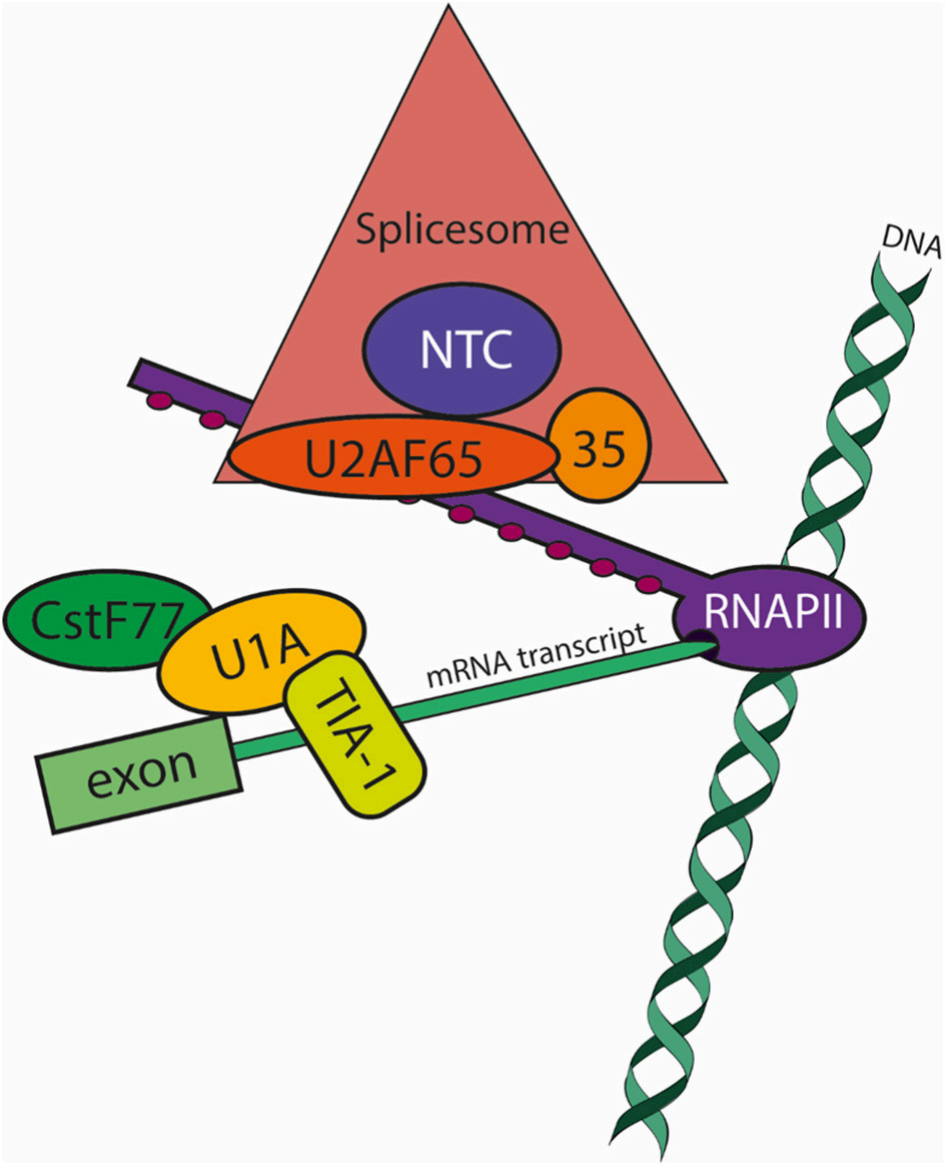
Co-transcriptional pre-mRNA processing in *E. histolytica*: focus on splicing factors. The model summarizes the data available to date (Valdés et al., 2014). During transcript elongation by RNA polymerase II (RNAPII; purple), Ser2 residues of the few heptapeptide repeats of its carboxy-terminal domain become phosphorylated (PSer2-CTD; maroon circles) and apt to recruit the spliceosomal (salmon triangle) and polyadenylation machineries. The large subunit of the U2 Auxiliary Splicing Factor U2AF65 (of 84 kDa in *E. histolytica*; orange oval) is a major player in pre-mRNA processing by tethering the spliceosome and the pre-mRNA (light green boxes) to RNAPII. U2AF65 interacts with the RNAPII-PSer2-CTD and with splicing factors conforming the Prp19 Complex (NTC; blue circle). The NTC regulates the formation and progression of essential spliceosome conformations required for the two steps of spacing. Splicing complex E formation occurs when the snRNP U1-A (yellow oval) binds to the 5′ss (splice site) and the splicing factor TIA-1/TIAR (yellow box) binds to the U-rich sequence just downstream the 5′ss. Splicing complex E also involves the 3′ss definition (not shown). When RNAPII releases the 3′ss from the transcription site, splicing factor 1 binds the branch site at the same time that U2AF65 binds the intron's polypyrimidine tract located between the branch site and the 3′ss; also simultaneously, the small subunit of U2AF (U2AF35, of 29 kDa in *E. histolytica*; pale orange circle) recognizes the 3′ss. The interaction of U2AF65 with splicing factor 1 and U2AF65 at the 3′ss and with the CTD of RNAPII ensures that U2AF65 also tethers the pre-mRNA to RNAPII. Finally, in addition to the previously reported interactions of RNAPII with the polyadenylation complex (vide infra), U1-A directly or indirectly interacts with the splicing complexes B-C, and more importantly with CstF77 (dark green oval), a member of the polyadenylation machinery.

Molecular evidence and cloning confirmed the presence of U2, U4, U5, and U6 snRNAs (Miranda et al., 1996; Davis et al., 2007), however no significant homology with eukaryotic U1 snRNAs has led to the conclusion that such small nuclear RNA is absent in *Entamoeba* (Dávila et al., 2008). Nonetheless bioinformatic analyses predicted the presence of the three U1 snRNP U1-A, U1-C, and U1-70k factors, suggesting that activation of the 5′ss might be due to direct interaction of snRNP proteins or by U6 snRNA-5′ss complementarity substitution as demonstrated in other systems (Kandels-Lewis and Seraphin, 1993; Förch et al., 2002; Rhode et al., 2006; Huang et al., 2012). *In vivo* expression of tag-cloned U1-A and cross-linking immunoprecipitation (CLIP) assays of nuclear proteins coupled to mass spectroscopy allowed the identification of at least 32 splicing factors in trophozoites (Table 1), namely U2, U4, and U5 snRNP, integral SmD1, SmD3, and SmF proteins; the U1 snRNP components and auxiliary factors U1-70k and TIA-1/TIAR; the U2 snRNP and related components U2-A', SF3a120, SF3a60/Prp9, SF3b1, SF3b3, U2AF65, and U2AF35; the U5 snRNP components Prp8 and Prp6 [which was previously identified and cloned (Hernandez-Rivas et al., 2000)]; the U6 snRNP integral components LSm2 and LSm5; two alleles of the U4/U6 di-snRNP component CP6; the U4/U6.U5 tri-snRNP components SAD1 and Prp38; and the nineteen complex (NTC) components Prp19, KIAA0560/Aquarius intron-binding spliceosomal factor, DDX5, and Abstrakt/DDX41 (Valdés et al., 2014) (Figure 1).

**Table 1:** Comparison of splicing factors in *Entamoeba histolytica* vs. human and yeast.

**Particle/class**	**Splicing factor**	**Remodeling**	***E. histolytica* protein**	**Locus^a^**	**UniProtKB**
Sm/LSm snRNP	SmD1		snRNP Sm D1	EHI_052090	B1N466
	SmD3		snRNP	EHI_163710	*C4M3V1*
	SmF		snRNP F	EHI_060400	C4M6J5
	LSm2		U6 snRNA-associated Sm-like LSm2	EHI_068580	C4LU49
	LSm5		hypothetical protein	EHI_076840	B1N3I3
U1 snRNP U1-related No U1 snRNA	U1A, HA-tag		U1 snRNP-specific protein	EHI_050780	C4LTU8
	U1-70K		U1 snRNP subunit	EHI_153670	C4LSE9
	p68	A-B (U1-5'ss)	EhDEAD20	EHI_096390	C4LWF2
	TIA-1/TIAR		RNA-binding protein TIA-1	EHI_056660	C4M9T1
U2 snRNP U2-related U2 snRNA	U2A'		leucine rich repeat protein	EHI_167290	C4MAF8
	SF3a120		splicing factor	EHI_058680	C4LWT7
	SF3a60/Prp9^PILC^		splicing factor 3A subunit 3	EHI_038600	C4LZP4
	SF3b1^PILC^		splicing factor 3B subunit 1	EHI_049170	C4MAD8
	SF3b3^PILC^		splicing factor 3b subunit 3	EHI_048160	C4M4A7
	U2AF65		U2 snRNP aux. fact. large subunit	EHI_098300	C4LXB3
	U2AF35		U2 snRNP auxiliary factor	EHI_192500	C4M1H0
	Prp43	ILS-disassembly	EhDExH9	EHI_184530	C4M6S9
	Prp43	ILS-disassembly	EhDExH13	EHI_090040	C4MA27
	Prp43	ILS-disassembly	EhDExH7	EHI_096230	C4LWD6
	Prp5	E-A (U2-3'ss)	EhDEAD3	EHI_013960	C4LXN8
U5 snRNP U5 snRNA	Snu114^PILC^	B-B^act^	U5 snRNP subunit	EHI_021380	B1N373
	Brr2^PILC^	B-B^act^; C-Post splicing	EhDExH10/U5 snRNP-specific 200kd	EHI_045170	C4LTD0
	Brr2^PILC^	B-B^act^; C-Post splicing	EhDExH1	EHI_131080	C4LXH6
	220K/Prp8^PILC^		splicing factor Prp8	EHI_060350	C4M6K0
	102K/Prp6		pre-mRNA splicing factor	EHI_093960	C4LYI7
	Prp28	A-B	EhDEAD4	EHI_021440	C4M058
U4/U6 snRNP	CPR6		peptidyl-prolyl cis-trans isomerase	EHI_020340	C4M7U6
U4/U6 snRNA	CPR6		peptidyl-prolyl cis-trans isomerase	EHI_125840	O15729
U4/U6·U5 tri-snRNP	65K/SAD1		ubiquitin C-term hydrolase	EHI_152110	C4LSP9
	Prp38		PRP38 family protein	EHI_000490	C4LZA0
Prp19C/IBC	Prp19^PILC^		WD domain containing protein	EHI_130870	C4LXF5
	CDC5^PILC^		myb-like DNA-binding	EHI_000550	C4LZA6
	Aquarius		regulator of nonsense transcripts	EHI_193520	C4M4V4
	Syf1^PILC^		Hypothetical protein	EHI_073300	C4LXJ5
	RBM22^PILC^		pre-mRNA splicing factor cwc2	EHI_126150	C4LW00
EJC	CWC22^#, PILC^		cell cycle control protein	EHI_093720	C4LYG3
	Sub2p/UAP56	E-A (U2-3'ss)	EhDEAD18	EHI_151600	C4LSK1
RES complex	MGC13125^PILC^		EF-hand calcium-binding	EHI_150550	C4M234
Complex B^act^	Prp2	B^act^-B*	EhDExH4	EHI_033720	C4M435
Complex C	Abstrakt		EhDEAD1	EHI_175030	C4LZM8
Step 2 factors	Prp22^PILC^	Post splicing-ILS	EhDExH3	EHI_077640	C4M8N6
	Prp16	C-Post splicing	EhDExH5	EHI_122790	C4M5M5

a *AmoebaDB. E. histolytica splicing factors, U snRNAs and components of the post-catalytic/intron lariat spliceosome complexes (PILS in superscript) were described by Miranda et al. (1996), Hernandez-Rivas et al. (2000), Davis et al. (2007), Dávila et al. (2008), Fourmann et al. (2013), and Valdés et al. (2014). Previously undetected additional Entamoeba PILS components (shaded) were identified in the ProteomeXchange repository PXD001080. # indicates that CWC22 is also part of the Prp19C. Transitions of spliceosome complexes remodeling by the respective DExH/D-box helicases are indicated (Liu, 2002; Marchat et al., 2008; Hahn et al., 2012; Wahl and Luhrmann, 2015)*.

Splicing E (early) complex formation involves 5′ss recognition by the U1 snRNP (Larson and Hoskins, 2017). However, the less conserved and poorly recognized (weak) 5′ss are activated by 5'ss-U1-C interactions or when TIA-1/TIAR binds to U-tracts localized in front of the 5′ss (Förch et al., 2002). Only U1-70k and TIA-1/TIAR were detected in the U1-A CLIP assays, therefore the most likely scenario for *Entamoeba* 5′ss activation involves direct interaction of U1-A/U1-70k with the 5′ss with the participation of TIA-1/TIAR bound to the U-rich most often spliced *Entamoeba* 5′ss (GUUUGUUU) (Hon et al., 2013) as described for weak 5′ss.

Because cross-linking was carried out with UV, the number of factors identified is limited but it represents all complexes formed during spliceosome assembly, first and second steps of splicing, disassembly, turnover, exon junction complex, and mRNA transport. Moreover, the presence of the core protein of the NTC, Prp19, and U2AF65, which interact with the PSer2 CTD of the large subunit of RNA pol II, ensure proper co-transcriptional activation of the spliceosome, splicing catalysis, termination factors recruitment, and extranuclear mRNA transport factors (David et al., 2011; Gu et al., 2013).

Also, DExH/D RNA helicases involved in the proofreading of the sequential steps of spliceosome assembly and catalysis were identified (Table 1): Prp5 and Sub2/UAP56 that facilitate E to A (pre-spliceosome) complex transition; p68, and Prp28, that promote transition from A complex to pre-catalytic (B) spliceosome; two alleles of Brr2, and Snu114, required for spliceosome activation (B to B^act^); Prp2, that catalytically activates the spliceosome (B^act^ to B^*^ complex); Prp16, that proof-reads the second step of splicing from the catalytic complex (C); the post-splicing complex helicase Prp22; and three alleles of the disassembly Prp43 helicase (Marchat et al., 2008; Valdés et al., 2014). The fact that all proofreading RNA helicases are present in *E. histolytica*, contrasts with the multiple splicing products repeatedly detected in deep RNA-seq data (Hon et al., 2013) indicating that additional cues control the splicing and alternative splicing of the majority of *Entamoeba* introns.

The components of the post-catalytic and of the intron lariat splicing complexes (collectively, PILC) have been recently identified (Fourmann et al., 2013). From our published data and the ProteomeXchange repository PXD001080, we identified the corresponding *E. histolytica* PILC factors: SF3a60/Prp9, SF3b1, SF3b3, Snu114, Brr2, 220K/Prp8, Prp19, CDC5, Syf1, RBM22, CWC22, MGC13125, and Prp22 (Table 1). Furthermore, the HA-tagged CLIP assays (Valdés et al., 2014) allowed the unprecedented identification of numerous messenger ribonucleoparticles factors, among them additional splicing-related factors, as well as components of the transcription (large subunit of RNA pol II, and various transcription factors), and polyadenylation (EhCstF64) machineries, evidencing the complexity of this co-transcriptional process (Figure 1).

Finally, since most *Entamoeba* pre-mRNAs are mono-intronic, and bioinformatic predictions and deep RNA-Seq data indicate that intron retention is the main route for alternative splicing (Davis et al., 2007; McGuire et al., 2008; Hon et al., 2013), splicing events impact both proteome expansion and gene expression regulation in this parasite.

### Intron lariat debranching enzyme

Intron lariat debranching enzyme, or Dbr1, is a member of the calcineurin-like metallophosphoesterases (MPEs) superfamily of binuclear metal-ion-center-containing enzymes that hydrolyse phosphomono-, phosphodi-, or phosphotri-esters in a metal-dependent manner. From bacteriophages to humans, the MPE domain is found in Mre11/SbcD DNA-repair enzymes, mammalian phosphoprotein phosphatases, acid sphingomyelinases, purple acid phosphatases, nucleotidases, and bacterial cyclic nucleotide phosphodiesterases. Despite this functional diversity, MPEs show a remarkably similar structural fold and active-site architecture composed of five sequence blocks that allow metal coordination in the conserved motif D[X]H[x]nGD[x]nGNH[D/E] [x]nH[x]nGH[X]H (Matange et al., 2015). Alanine scanning assays identified the yeast Dbr1 RNA debranching active-site *in vivo* and *in vitro*, which in *E. histolytica* correspond to residues Cys14, His16, Asp45, Asn90, His91, His180, His230, and His232; in all debranching enzymes in nature, cysteine substitutes an aspartic acid residue in position 14 (Matange et al., 2015; Schwer et al., 2016).

Compared to different Dbr1 orthologs, *E. histolytica* Dbr1 is the shortest protein due to its truncated C-terminus; i.e., it is ≈50 residues shorter that *S. cerevisiae* Dbr1. Thanks to its small size, *Entamoeba* Dbr1 has been able to co-crystalize with synthetic branched RNAs and analogs, providing insights of enzyme-lariat interactions. Initial structural studies confirmed the MPE β metal binding pocket (Montemayor et al., 2014) however there was no metal in the A pocket. Subsequent structures revealed the 2nd metal ion in the A pocket (Clark et al., 2016; Ransey et al., 2017). Importantly, this approach identified within the MPE the lariat recognition loop (LRL) whose recognition module, unique to Dbr1 enzymes, comprises residues Ile132, Tyr133, Glu138, Pro141, Tyr144, and Pro148. Dbr1-analogs co-crystals showed that Dbr1 prefers 2′ purines in the branch, although the pocket could accommodate pyrimidines. In addition, there are few sequence-specific interactions at the BS, confirming recognition of atypical BS. Finally, the interactions between RNA and the LRL are stabilized by secondary contacts between residues 141–144 of the LRL and residues Phe292, Pro293, and Phe337 of the carboxy-terminal domain (CTD) of Dbr1; and intricate hydrogen bonds centered in Arg158 aid to stabilize further the conformation of the LRL (Montemayor et al., 2014). The structural data was confirmed by Dbr1 activity *in vivo*. Whereas, *E. histolytica* wild type Dbr1 was able to complement *Saccharomyces cerevisiae* Dbr1-deletant strains relieving intron lariat accumulation, none of the constructs carrying Cys14Ala/Ser substitutions or 141-146Ala substitutions, or CTD or LRL deletions relieved intron lariat accumulation (Montemayor et al., 2014). The presence of *E. histolytica* Dbr1 in intron large post-spliceosomal complexes along with U2, U5 and U6 snRNP components, and the proteins Ntr1/TFIP11 and Prp43 (Yoshimoto et al., 2009) or the Drn1/Ygr093w protein that transiently binds Dbr1 to post-spliceosomal complexes is still unproven (Garrey et al., 2014), although our previous findings point to this possibility (Valdés et al., 2014).

## Understanding polyadenylation in *E. histolytica*

### Cis-elements for pre-mRNA 3′ end formation

One of the first reports about mRNA polyadenylation in *E. histolytica* was published in 1993 and describes the existence of a putative polyadenylation motif TAATT and a 12 pyrimidine stretch in the 3′UTR of parasite genes (Bruchhaus et al., 1993). Then, other groups showed that alternative polyadenylation sites and poly(A) tail size represent efficient postranscriptional mechanisms for gene expression regulation (Urban et al., 1996; López-Camarillo et al., 2003). But the publication of the first version of the *E. histolytica* genome sequence in 2005 (assembly of ~23 Mb that predicted 9938 coding genes comprising 49% region of the genome) (Loftus et al., 2005) represented the critical step to identify motifs in the mRNA 3′UTR and the polyadenylation machinery in this parasite.

**Figure 2:**
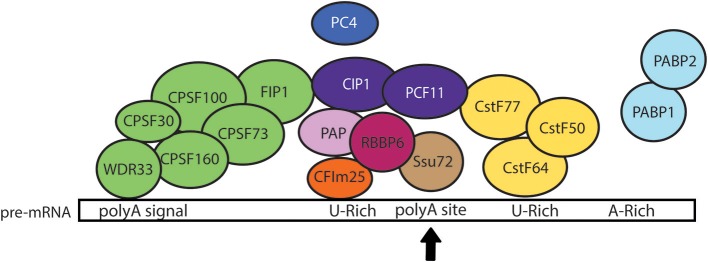
Model of the pre-mRNA 3′ processing complex in *E. histolytica*. The core 3' processing complex is composed of four main complexes: CPSF (green), CstF (yellow), CFIm (orange), and CFIIm (purple) that bind *cis*-elements within pre-mRNA 3′ UTR. Based on the current knowledge in other eukaryotic cells, we hypothesize that the WDR33 subunit of CPSF complex recognizes the polyadenylation signal (polyA signal) located upstream of the cleavage site (polyA site marked here with a black arrow), while CstF64 recognizes the U-rich downstream element. We also propose that the binding of CFIm25 to the upstream U-rich motif promotes the recruitment of CFIIm subunits (CIP1 and PCF11) and interactions between CPSF and CstF, allowing the RNA cleavage by CPSF73 and the poly(A) tail synthesis by PAP. Additional factors, such as FIP1, PC4, RBBP6, Ssu72, and PABP, would also contribute to the regulation of cleavage/polyadenylation reaction (López-Camarillo et al., 2005). Interactions between EhCFIm25 and EhPAP have been experimentally confirmed *in vitro* (Pezet-Valdez et al., 2013). Interestingly, the interaction of EhCstF77 with U1-A provided the first evidence for a link between mRNA polyadenylation and splicing in *Entamoeba* (Valdés et al., 2014).

A small-scale *in silico* analysis of cDNA and genomic sequences revealed that *E. histolytica* 3′ UTRs contain three conserved motifs: (i) the consensus UA(A/U)UU polyadenylation signal or variants located 10–30 nt upstream the poly(A) site, (ii) the U-rich tract located 1–30 nt upstream the poly(A) site, and (iii) a U-rich element located 3–30 nt downstream the poly(A) site (López-Camarillo et al., 2005). Computational examination of a larger number of cDNA and genomic sequences confirmed this molecular array and suggested the presence of an additional distal A-rich element (Figure 2) (Zamorano et al., 2008). Study of the alternative usage of poly(A) sites using RNA-Seq indicated that microheterogeneity in poly(A) sites is likely to be stochastic in *E. histolytica* and only a small fraction of alternative polyadenylation isoforms appeared to be genuine (Hon et al., 2013). Interestingly, genes with alternative poly(A) sites may have a large impact on global gene expression in *E. histolytica* since most of them participate in DNA condensation, DNA binding, translation, splicing, mRNA binding, protein folding and protein transport; other genes are related to signaling, oxidation/reduction, calcium ion binding, cell cycle, and intracellular transport. Indeed, the upstream shift in poly(A) site selection resulting from the silencing of the polyadenylation factor EhCFIm25, was confirmed for thioredoxin and 60S ribosomal protein L7 transcripts and related to specific phenotypical changes and parasite death (Ospina-Villa et al., 2017).

### Polyadenylation factors

Analyses of the 9938 coding genes predicted in the first version of the genome indicated that *E. histolytica* has genes that encode proteins with homology to the majority of polyadenylation factors described in human and yeast (Table 2): the cleavage and specificity factor (CPSF160, 100, 73, and 30), the cleavage stimulating factor (CstF77, 64, and 50), the 25 kDa subunit of the cleavage factor Im (CFIm) and both C1P1 and PCF11 subunits of CFIIm, as well as FIP1, poly(A) polymerase (PAP), poly(A) binding protein (PABP), RBBP6 (Mpe1 in yeast), WDR33 (Psf2 in yeast), PNAS-120 (Ssu72 in yeast), and PC4 (Sub1 in yeast) (Figure 2) (López-Camarillo et al., 2005, 2014). In human cells, WDR33 and CPSF30 are the CPSF subunits that binds the polyadenylation signal (Chan et al., 2014; Schonemann et al., 2014). CPSF73 is the endonuclease responsible for RNA cleavage (Mandel et al., 2006). CstF77 interacts with CPSF160, promoting their cooperative RNA binding during the assembly process (Murthy and Manley, 1995). CFIm25 regulates the selection of distal poly(A) sites, contributes to the recruitment of polyadenylation factors and is necessary for poly(A) tail synthesis (Brown and Gilmartin, 2003; Kubo et al., 2006). Although CFIIm subunits are the less characterized polyadenylation factors, evidence suggest that Pcf11 is required for degradation of the 3′ product following cleavage (West and Proudfoot, 2008), while Clp1 interacts with both CPSF and CFIm and likely tethers them to CFIIm (de Vries et al., 2000). PAP is responsible for the addition of the polyadenosine tail (Raabe et al., 1991) and its activity is accelerated by PABP (Wahle, 1991). RBBP6 associates with other core polyadenylation factors through its unusual ubiquitin-like domain and modulates expression of mRNAs with AU-rich 3' UTR (Di Giammartino et al., 2014). PC4 (Sub1 in yeast) associates with the polyadenylation factor CstF64 to modulate transcription termination and polyadenylation initiation (Calvo and Manley, 2001, 2003).

**Table 2:** Polyadenylation factors in human and *E. histolytica*.

***H. sapiens***		***E. histolytica***		
**Protein**	**Access number**^a^	**Locus**^b^	**Protein**	**Access number**^a^
**CLEAVAGE AND POLYADENYLATION SPECIFIC FACTORS**				
CPSF160	Q10570	EHI_160110	CPSF160	C4M386
CPSF100	Q9P2I0	EHI_033130	CPSF100	C4M6Y0
CPSF73	Q9UKF6	EHI_136700	CPSF73	C4M297
CPSF30	O95639	EHI_067580	CPSF30	C4M9G4
FIP1	Q6UN15	EHI_052180	FIP1	C4M765
WDR33	Q9C0J8	EHI_170080	WDR33	C4M1D0
**CLEAVAGE STIMULATING FACTORS**				
CstF77	Q12996	EHI_098370	CstF-77	B1N2W3
CstF64	P33240	EHI_151900	CstF-64	C4LSN8
CstF50	Q05048	EHI_152770	CstF-50	C4LSW0
**CLEAVAGE FACTORS IM**				
CFIm68	Q16630	–	–	–
CFIm59	Q8N684	–	–	–
CFIm25	O43809	EHI_077110	CFIm25	C4M2T1
**CLEAVAGE FACTORS IIM**				
CIP1	Q92989	EHI_008100	CIP1	C4LYE5
PCF11	O94913	EHI_045130	PCF11	C4LTC6
**OTHER POLYADENYLATION FACTORS**				
PAP-B	Q9NRJ5	EHI_012040	PAP	Q51D88
Ssu72	Q9BZS6	EHI_027340	Ssu72	C4M1T3
PC4	P53999	EHI_192520	PC4	C4M1H2
PABP1	P11940	EHI_198750	PABP1	C4LWS1
		EHI_033250	PABP2	C4M6Y2
RRBP6	Q7Z6E9	EHI_014000	RRBP6	C4LXP1
Symplekin	Q92797	–	–	–

a UniProtKB;

b *AmoebaDB. Data about parasite proteins were obtained from (López-Camarillo et al., 2005, 2014)*.

The presence of these proteins in *E. histolytica* suggests that 3′ end processing of parasite mRNA could be performed as it has been described in other eukaryotic cells. Accordingly, EhPAP has the conserved PAP central catalytic domain with the three invariant aspartate residues involved in nucleotide transfer and the F/YGS motif responsible for ATP binding, confirming that it belongs to the polymerases-like superfamily of nucleotidyl transferases (García-Vivas et al., 2005). In the predicted three-dimensional model, both functional domains fold as a U-shaped structure that likely orients the incoming ATP and RNA molecules for poly(A) tail extension. In agreement with its expected role in the poly(A) tail synthesis, EhPAP was able to bind RNA 3′ end although it has a divergent RNA-binding domain (RBD). Moreover, it was located in punctuate nuclear *foci* and in cytoplasmic dots, as it has been described for other polyadenylation factors (García-Vivas et al., 2005; López-Camarillo et al., 2007).

As the human homolog, the EhCFIm25 protein is an unconventional Nudix protein lacking three of the four conserved E residues and the last G residue of the Nudix box, which suggests that it is not able to cleave RNA. Despite the absence of a classical RBD, EhCFIm25 is able to bind the 3′ UTR of *E. histolytica* transcripts and this interaction involves the participation of the conserved Leu135 and Tyr236 residues (Ospina-Villa et al., 2015). EhCFIm25 recognizes the GUUG motif in mRNA 3′end, while the human protein binds the UGUA sequence; consequently, aptamers containing this motif specifically identify the parasite protein, suggesting that they could be used as molecular tools for diagnosis or therapeutic purposes (Ospina-Villa et al., 2018). Interestingly, EhCFIm25 interacts with EhPAP (Pezet-Valdez et al., 2013), which indicates that it may be introduced into the processing complex in the early steps of the cleavage/polyadenylation reaction. EhCFIm25 controls the selection of the distal poly(A) site during mRNA 3′ end formation; consequently, its silencing by dsRNA or its blocking by aptamers alter parasite proliferation and virulence, demonstrating for the first time the relevance of polyadenylation factors as biochemical targets in *E. histolytica* (Ospina-Villa et al., 2017, 2018). Other works have also recently reported that targeting polyadenylation factors represents a valuable strategy for the control of the protozoan parasites *Trypanosoma brucei* (CPSF-30), *Toxoplama gondii*, and *Plasmodium falciparum* (CPSF-73) (Hendriks et al., 2003; Sidik et al., 2016; Palencia et al., 2017; Sonoiki et al., 2017).

EhPC4 is the homolog of the human positive coactivator 4, a multifunctional protein that establishes an important link between transcription and polyadenylation. On one hand, its binding to promoters facilitates the recruitment of transcription factors to stimulate the pre-initiation complex assembly (Conesa and Acker, 2010); on the other hand, its interaction with EhCstF64 avoids premature transcription termination and polyadenylation initiation until the polyadenylation motifs have been transcribed (Calvo and Manley, 2001). Moreover, it mediates chromatin organization and heterochromatin gene silencing by interacting with histones H3 and H2B (Das et al., 2006, 2010). As homologous proteins, the EhPC4 protein contains a single-strand DNA (ssDNA) binding region whose residue K127 is required for DNA interaction, and a dimerization domain in the so-called PC4 domain at the C-terminus (Hernandez de la Cruz et al., 2014). Interestingly EhPC4 and its potential partner, EhCstf-64, were significantly up-regulated in virulent trophozoites (Santi-Rocca et al., 2008). Consistently, the overexpression of EhPC4 induced the modulation of proteins with key functions in cytoskeleton dynamics, cell migration and invasion in trophozoites. Among them, the up-regulation of a 16-kDa actin-binding protein (EhABP16) which is a putative member of the cofilin/tropomyosin family involved in actin polymerization was associated with an increase in parasite migration of trophozoites and destruction of human SW480 colon cells, confirming that EhPC4 has an impact on parasite virulence (Hernandez de la Cruz et al., 2014). On the other hand, the overexpression of EhPC4 significantly increased cell proliferation, DNA replication and DNA content of trophozoites, promoting the formation of giant multinucleated trophozoites. EhPC4 modulates the expression of genes involved in carbohydrate and nucleic acid metabolism, chromosome segregation and cytokinesis, evidencing the relevance of this factor in polyploidy and genome stability in *E. histolytica* (Hernández de la Cruz et al., 2016). The role of EhPC4 in mRNA 3′end formation and its relevance for the events mentioned above remain to be investigated.

## Molecular events for mRNA decay in *E. histolytica*

### mRNA degradation machineries

**Figure 3:**
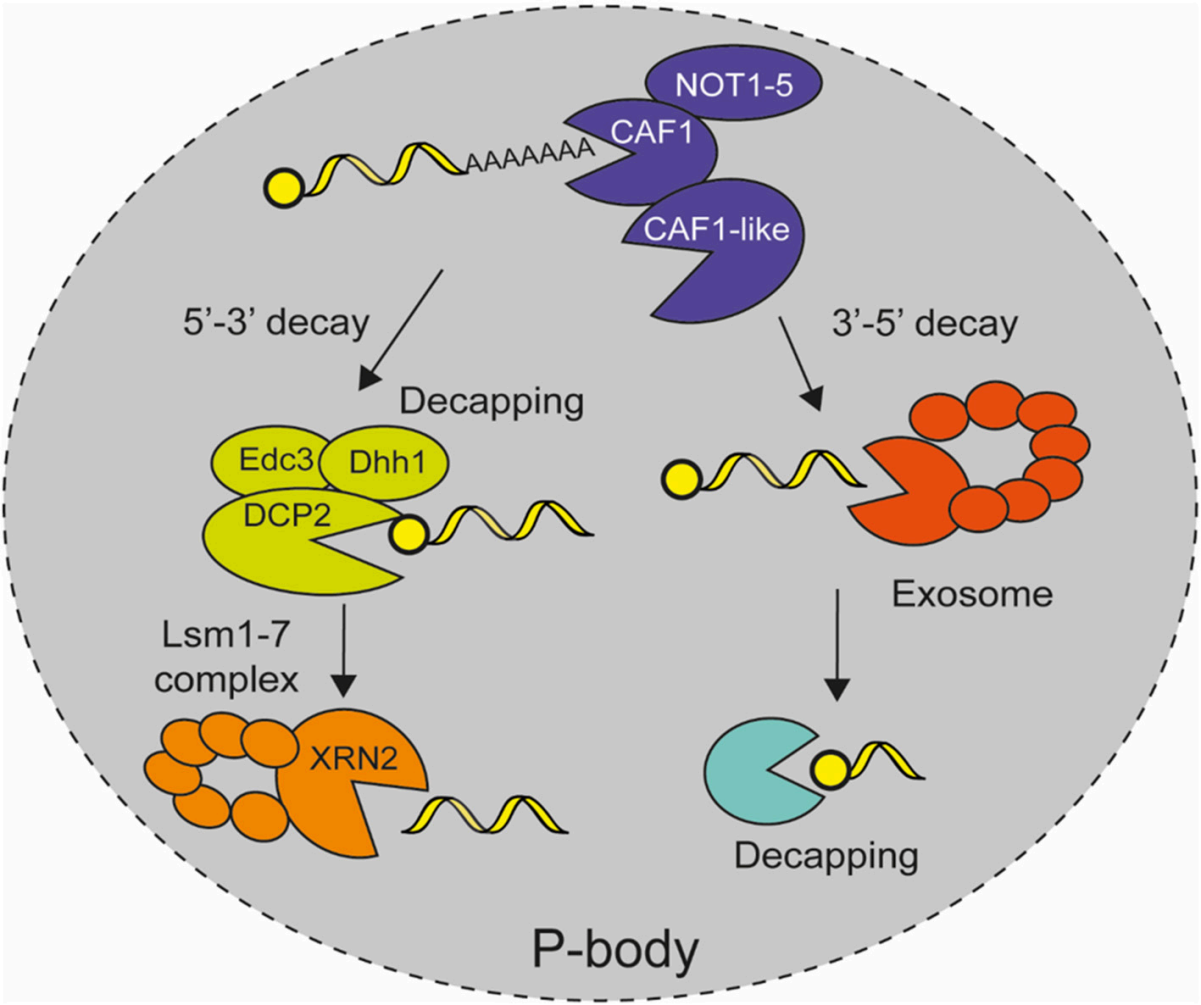
Model for mRNA decay in *E. histolytica***.** After mature mRNAs are exported to the cytoplasm where they are eventually translated in a regulated manner, they are later degraded in cytoplasmic P-bodies like structures. The mRNA degradation pathway starts with transcript deadenylation by the CAF1/NOT complex. Then, DCP2 mRNA decapping enzyme removes the cap structure from the 5′ end of the mRNA with the help of accessories proteins Edc3 and Dhh1, which allows 5′-3′ exoribonuclease XRN2 with Lsm protein complex to degrade the rest of mRNA body. Alternatively, the deadenylated transcript is degraded in a 3′-5′ direction by the catalytic subunit Dis3 of the exosome complex and the cap structure is eliminated by DCPs protein (López-Rosas et al., 2012, 2014).

*E. histolytica* has most of the factors that are involved in mRNA degradation in eukaryotic cells, including proteins involved in deadenylation, decapping, and exonuclease activity, but it lacks several components (Table 3) (López-Rosas et al., 2012). The reduced mRNA deadenylation machinery includes the CAF1/NOT complex with the five NOT proteins and the poly-A specific ribonucleases CAF1 and CAF1-like, but the carbon catabolite repressor 4 (CCR4) described in yeast and human, as well as PAN2, PAN3, and PARN deadenylases, are missing (Figure 3). EhCAF1 is a ribonuclease D family member, having the CAF1 nuclease domain and the conserved DEDD residues (D_84_E_86_D_206_D_276_) that are important for 3′ to 5′ exonuclease activity in homologous proteins (Daugeron et al., 2001). Consequently, EhCAF1 is a functional deadenylase that binds 3′UTR and degrades the poly(A) tail of parasite transcripts in *in vitro* assays (López-Rosas et al., 2014).

**Table 3:** Comparison of mRNA decay machineries between human and *E. histolytica*.

***H. sapiens***		***E. histolytica***			
**Protein**	**Access number**^a^	**Locus**^b^	**Protein**	**Access number**^a^	**References**
**DECAPPING FACTORS**					
DCP2	Q8IU60	EHI_058810	EhDCP2	C4M5G6	López-Rosas et al., 2012
Lsm1	O15116	EHI_188020	EhLsm 1	B1N3A8	
Lsm2	Q9Y333	EHI_068580	EhLsm2	C4LU49	
Lsm3	P62310	EHI_151310	EhLsm3	C4LSH4	
Lsm4	Q9Y4Z0	EHI_049370	EhLsm4	C4LUD9	
Lsm5	Q9Y4Y9	EHI_076840	EhLsm5	B1N3I3	
Lsm6	P62312	EHI_188130	EhLsm6	C4M187	
Lsm7	Q9UK45	EHI_025840	EhLsm7	C4M939	
Edc3	Q96F86	EHI_198940	Ehedc3	C4LWU0	
Dhh1	P26196	EHI_093900	Ehdhh1	C4LYI1	
XRN2	Q9H0D6	EHI_133330	EhXRN2	C4MB40	
**DEADENYLATION FACTORS**					
CAF1	Q9UIV1	EHI_ 048150	EhCAF1	Q56AY2	López-Rosas et al., 2012, 2014
CALIF	Q9UFF9	EHI_039000	EhCAF1-like	C4LZS1	López-Rosas et al., 2012
NOT1	A5YKK6	EHI_200810	EhNOT1	C4M3Y6	
NOT2	Q9NZN8	EHI_044180	EhNOT2	C4M790	
NOT3	O75175	EHI_119550	EhNOT3	C4M7Y3	
NOT4	O95628	EHI_080710	EhNOT4	C4M9N9	
**EXOSOME FACTORS**					
RRP4	Q13868	EHI_163510	EhRRP4	C4M3T2	López-Camarillo et al., 2014
RRP6	Q01780	EHI_021400	EhRRP6	C4M054	
RRP40	Q9NQT5	EHI_004770	EhRRP40	C4M073	
RRP41	Q9NPD3	EHI_040320	EhRRP41	C4M2G1	
RRP42	Q15024	EHI_000580	EhRRP42	C4LZA9	
RRP43	Q96B26	EHI_188080	EhRRP43	C4M182	
RRP46	Q9NQT4	EHI_086520	EhRRP46	C4M8Y9	
MTR3	Q5RKV6	EHI_126330	EhMTR3	C4LW17	
DIS3	Q9Y2L1	EHI_160720	EhDIS3	C4MAJ9	
**NON-SENSE MEDIATED DECAY FACTOR**					
UPF1	Q92900	EHI_035550	EhUpf1	C4LXY5	López-Rosas et al., 2012
**RNA INTERFERENCE FACTORS**					
AGO2	Q9UKV8	EHI_186850	EhAGO2-1	C4LVQ2	Zhang et al., 2008
–	–	EHI_125650	EhAGO2-2	C4LVV2	
–	–	EHI_177170	EhAGO2-3	C4LY31	
–	–	EHI_139420	EhRdRP	C4M6W7	
–	–	EHI_179800	EhRdRP	C4M631	
DICER	Q9UPY3	EHI_068740	EhRNAseIII	C4LU64	Abed and Ankri, 2005; Zhang et al., 2008

a UniProtKB;

b *AmoebaDB*.

Although capping has not been described in *E. histolytica*, bioinformatics analyses revealed the existence of a decapping complex that is formed by the catalytic subunit EhDCP2, and EhXRN2, EhLSM1–6, EhEDC3, and EhDHH1 as decapping associated proteins, whereas it also includes DCP1, SCD6, PAT1, and LSM7 in yeast and animals (Table 3; Figure 3) (López-Rosas et al., 2012). EhXRN2 and EhDCP2 have the typical architecture of homologous proteins. Notably, EhXRN2 has the XRN_N nuclease domain and the internal tower domain with the active site motif KX_2_QQX_2_RR, which is critical for ribonuclease function (Xiang et al., 2009). EhDCP2 has the conserved DCP2 box A domain and the conserved nudix Box (GX_5_EX_7_REUXEEXGU) that are both responsible for cap structure removal (She et al., 2008). In eukaryotic cells, the elimination of the 5′ cap compromises mRNA to 5′ to 3′ exonucleolytic decay, apparently in an irreversible way, hence, decapping activity is tightly regulated (Li and Kiledjian, 2010). The heptameric Lsm1–7 complex associates with the 3' end of deadenylated mRNAs and promotes decapping (Tharun et al., 2000; Tharun and Parker, 2001). The activity of the decapping enzyme is stimulated by accessory proteins, such as Edc proteins, the DExD/H-box RNA helicases Dhh1 and Pat1 (Bonnerot et al., 2000; Schwartz et al., 2003).

*E. histolytica* contains seven exosome encoding genes including Rrp41, Rrp43, Rrp46, Mtr3-Rrp42 and the catalytic subunit Dis3, as well as accessory stabilizing Rrp4, and Rrp40 proteins; but it lacks Rrp45 and Csl4 genes (Table 3; Figure 3) (López-Rosas et al., 2012). The EhRRP41 protein colocalizes and physically interacts with EhL-PSP, which also interacts and colocalizes with the EhCAF1 deadenylase. But the fact that EhRRP41 did not coimmunoprecipitate with EhCAF1, suggests the existence of two EhL-PSP-containing complexes. The colocalization of exosome factors (EhRrp41) with EhCAF1 and EhL-PSP in trophozoites showed novel interactions between mRNA degradation protein and suggests the existence of cooperative interactions between mRNA decay machineries in *E. histolytica* (López-Rosas et al., 2014). In yeast and human, the nine subunits of the exosome complex form a ring structure in which Dis3 (RRP44) is the key player in mRNA turnover being the catalytic subunit responsible for exonucleolytic and endonucleolytic activities in the 3′-5′ decay of deadenylated transcripts in cytoplasm (Ibrahim et al., 2008). Additionally, the nuclear exosome is involved in 3′-end trimming of rRNA, snRNA, and snoRNA, as well as mRNA surveillance and degradation of cryptic unstable transcripts (Parker and Song, 2004).

*E. histolytica* genome also contains genes for components of the NMD and RNA interference (RNAi) pathways, namely three Ehupf genes (López-Rosas et al., 2012), as well as two EhRdRP, one EhRNAseIII and three EhAGO2 proteins (Abed and Ankri, 2005; Zhang et al., 2008, 2011), respectively (Table 3). The absence of DICER and GW182 homologs suggests that RNA interference may use DICER-independent mechanisms in *E. histolytica* (Zhang et al., 2011). Pompey et al. (2015) recently showed that EhRNAseIII is able to cleave dsRNA to generate shorter fragments in a heterologous system. This suggests that EhRNAseIII in conjunction with other amoebic factors might reconstitute an active DICER-like complex. Congruently, numerous reports involving gene-silencing assays confirmed the functionality of the RNAi pathway in *E. histolytica*.

### P-body-like structures

Several experiments suggest that mRNA decay reactions, namely deadenylation, decapping, and 5′-exonucleolytic decay, take place in microscopically detectable cytoplasmic P-bodies like structures in *E. histolytica* (López-Rosas et al., 2012), as it has been described in other eukaryotic cells (Sheth and Parker, 2003). The EhCAF1 deadenylase, EhXRN2 exoribonuclease and EhDCP2 decapping proteins, as well as the EhAGO2-2 protein, were detected in cytoplasmic foci in immunofluorescence and confocal microscopy experiments (López-Rosas et al., 2012). Additionally biochemical analysis revealed that EhCAF1 co-immunoprecipitated with EhXRN2, thus linking deadenylation to 5'-to-3' mRNA degradation. Interestingly, these cytoplasmic structures also contain polyadenylated transcripts and dsRNA, which is congruent with their role in RNA decay. Moreover their formation depends on the presence of active transcription and translation (López-Rosas et al., 2012), as well as cellular stress, such as DNA damage, heat shock, and nitric oxide (López-Rosas et al., 2014), which make them *bona fide* P-body structures (Figure 3). Altogether, these data suggest that, as in human cells, the accumulation of transcripts in cytoplasmic P-bodies like structures for silencing or decay, represents a key regulatory process for gene expression regulation in response to specific conditions or signals in *E. histolytica*.

## Conclusion

Besides the evolutionary distance between *E. histolytica* and its human host, the screening of parasite genome sequences and the functional characterization of specific factors, revealed that molecular mechanisms regulating mRNA processing and degradation seem to be roughly similar in both organisms. Several subtle differences exist, but canonical factors involved in splicing, polyadenylation and decay are generally conserved in this primitive eukaryote, which highlights that these events are key players for gene expression regulation in eukaryotic cells. The study of a larger number of factors involved in splicing, polyadenylation or mRNA degradation remains to be addressed to elucidate all the relationship among these reactions. In addition to contribute to the better understanding of posttranscriptional regulation in *E. histolytica*, the characterization of these factors and events may also lead to the identification of a biochemical target involved in various mRNA processing pathways, whose inhibition would have a massive impact on parasite survival. On the other hand, recent data indicated the potential of factors involved in polyadenylation as biochemical targets for parasite control, which may open the way for the design of new molecules for the control of this parasitic disease. In this context, the results of the proof-of-concept study in *E. histolytica* may promote the use of aptamers to control *E. histolytica* during the development of amoebiasis or to eradicate residual trophozoites during antibiotic treatment. Further experiments are required to confirm their affinity, evaluate their effect *in vivo* and improve their bioavailability.

## Author contributions

JV-F, IL-R, CL-C, ER-M, and JO-V reviewed data about mRNA splicing, polyadenylation and decay, respectively. JO-V designed the figures. CL-C and LM designed the review organization, revised and integrated the different parts of the manuscript.

### Conflict of interest statement

The authors declare that the research was conducted in the absence of any commercial or financial relationships that could be construed as a potential conflict of interest. The handling Editor declared a shared affiliation, though no other collaboration, with one of the authors JV-F.
